# Activation of SphK2 contributes to adipocyte-induced EOC cell proliferation

**DOI:** 10.1515/med-2022-0422

**Published:** 2022-01-31

**Authors:** Lan Dai, Chen Wang, Wenjing Wang, Keqi Song, Taiyang Ye, Jie Zhu, Wen Di

**Affiliations:** Department of Obstetrics and Gynecology, Ren Ji Hospital, School of Medicine, Shanghai Jiao Tong University, Shanghai 200127, China; Department of Cell Biology, Shanghai Key Laboratory of Gynecologic Oncology, Ren Ji Hospital, School of Medicine, Shanghai Jiao Tong University, Shanghai 200127, China

**Keywords:** epithelial ovarian cancer, adipocytes, sphingosine kinase 2, proliferation

## Abstract

Epithelial ovarian cancer (EOC) is the leading cause of deaths due to cancer in women. Adipocytes have been suggested to play a key role in the stimulation of EOC growth. However, the mechanisms underlying the adipocyte-induced EOC proliferation remain undefined. Here, we provide the first evidence that adipocytes induce the activation of sphingosine kinase (SphK) 2 in EOC, which represents a novel pathway that mediates the adipocyte-induced EOC growth. SphK2 inhibition in EOC cells led to a remarkable inhibition of the adipocyte-induced cell proliferation. Moreover, the adipocyte-induced SphK2 activation in EOC cells was extracellular signal-regulated protein kinases (ERK) dependent. Furthermore, silencing SphK2 in EOC significantly inhibited the adipocyte-induced expression of phospho-ERK and c-Myc, two crucial players in EOC growth. Collectively, the current study unraveled a previously unrecognized role of SphK2 in the adipocyte-induced growth-promoting action in EOC, suggesting a novel target for EOC treatment.

## 1 Introduction

Epithelial ovarian cancer (EOC) is the deadliest gynecological malignancy [1]. Most EOC patients are found to have tumors in an advanced stage at initial diagnosis, which may lead to high disease mortality [2]. Therefore, understanding the mechanisms that regulate EOC growth may have an important impact on the outcome of this fatal cancer. EOC growth is particularly affected by adipocytes [3,4,5,6,7]. EOC cells have a predilection to proliferate in the omentum, an organ primarily composed of adipocytes [3,6,7]. *In vitro*, coculture of EOC cells with adipocytes can promote the proliferation of EOC. *In vivo*, subcutaneous injection of EOC cells with adipocytes into nude mice can produce tumors larger than those produced using EOC cells alone [3]. Moreover, as a source of various adipokines, adipocytes can provide high-energy metabolites and a series of factors for EOC growth [5,8]. However, the molecular mechanisms responsible for the growth-promoting effect of adipocytes in EOC remain unclear.

Sphingolipid metabolism dysregulation is often associated with cancer initiation and progression [9,10]. Sphingosine kinases (SphKs), the key enzymes controlling sphingolipid metabolism, are emerging as exploitable targets for cancer therapy [10,11]. SphKs contain two isoforms, SphK1 and SphK2. SphK1 has been reported to be involved in many aspects of EOC progression [12,13]. SphK1, which was overexpressed in EOC tissue [14], was inversely correlated with overall survival (OS) in EOC patients [15]. Moreover, elevated SphK1 levels were associated with EOC growth [16], metastasis [14], angiogenesis [17], and chemotherapeutic resistance [18]. Furthermore, knockout of SphK1 significantly blocked the EOC progression [19]. Unlike extensively studied SphK1, the roles of SphK2 are controversial and still poorly characterized. Initially, SphK2 was considered to be a proapoptotic protein because overexpression of SphK2 promoted tumor apoptosis [20]. However, it was subsequently found to be a prosurvival factor, as inhibition of SphK2 suppressed the tumor growth [21]. Our earlier studies showed that SphK2 is mainly located in the nucleus of ovarian cancer cells [15,17] and is potentially involved in the regulation of gene activation [22,23]. SphK2 activation was a prognostic indicator of OS in EOC patients [15]. In addition, knockdown of SphK2 arrested the cell cycle progression and inhibited the EOC cell proliferation both *in vitro* and *in vivo* [23]. Moreover, inhibition of SphK2 was shown to sensitize EOC to paclitaxel [24]. Although SphK2 is an important signaling enzyme in EOC progression, its regulatory mechanisms are far from clarified. In this study, we provide the first evidence that adipocytes are capable of activating SphK2 and unravel a previously unrecognized role of SphK2 in the adipocyte-induced growth-promoting action in ovarian cancer.

## 2 Materials and methods

### 2.1 Reagents and antibodies

Antibodies against SphK2 (ab264042, rabbit), c-Myc (ab32072, rabbit), and glyceraldehyde-3-phosphate dehydrogenase (GAPDH) (ab8245, mouse) were purchased from Abcam (Cambridge, MA, USA). Antibodies against ERK1/2 (4696, rabbit) and phospho-ERK1 (Thr202/Tyr204)/ERK2 (Thr185/Tyr187) (4370, rabbit) were ordered from Cell Signaling Technology (Danvers, MA, USA). Antibodies against phosphor-SphK2 (Thr578) (SP4631, rabbit) were purchased from ECM Biosciences (Versailles, KY, USA). U0126, ABC294640, insulin, dexamethasone, and 3-isobutyl-1-methylxanthine were ordered from Sigma-Aldrich (St. Louis, MO, USA).

### 2.2 Cell lines and culture conditions

The human EOC cell line A2780 was obtained from the China Center for Type Culture Collection. SKOV3 cells were purchased from American Type Culture Collection. These two EOC cell lines were cultured in Dulbecco's Modified Eagle Medium (DMEM) (Invitrogen, Carlsbad, CA, USA) supplemented with 10% fetal bovine serum (Invitrogen) and 1% antibiotics. The murine 3T3-L1 preadipocyte cell line was ordered from the Cell Bank of the Chinese Academy of Sciences (Shanghai, China). 3T3-L1 cells were cultured in DMEM supplemented with 10% calf serum and 1% antibiotics. 3T3-L1 preadipocytes were induced into mature adipocytes by treatment with insulin, dexamethasone, and 3-isobutyl-1-methylxanthine as described earlier [25]. To make adipocyte-conditioned medium (Adi-CM), mature adipocytes were cultured with serum-free medium (SFM) for 24 h after being washed twice with phosphate buffered saline. Adi-CM was then collected and filtered.

### 2.3 Small interfering RNA (siRNA) and transient transfection

The chemically synthesized siRNAs targeting human SphK2 (5′-AACCUCAUCCAGACAGAACGA-3′) and the control siRNA (5′-AAUUCUCCGAACGUGUCACGU-3′) were ordered from GenePharma (Shanghai, China) [17]. SiRNA transfection was performed by using Lipofectamine (Invitrogen). The levels of SphK2 were detected by reverse transcription-polymerase chain reaction (RT-PCR) and Western blot 24–48 h after transfection.

### 2.4 Real-time RT-PCR

RNA was extracted by using TRIzol Reagent (Invitrogen). The mRNA levels were detected by using Synergy Brands Green RT-PCR and calculated by the 2^−ΔΔCt^ method. Primers were as follows: SphK2, 5′-GGTTGCTTCTATTGGTCAATCC-3′ (forward) and 5′-GTTCTGTCGTTCTGTCTGGATG-3′ (reverse); and GAPDH, 5′-TGCACCACCAACTGCTTAGC-3′ (forward) and 5′-GGCATGGACTGTGGTCATGAG-3′ (reverse).

### 2.5 Western blot analysis

Western blot analysis was performed as described earlier [26]. Briefly, cells were harvested and lysed with RIPA buffer plus protease inhibitors. The proteins were resolved by sodium dodecyl sulfate polyacrylamide gel electrophoresis and transferred to polyvinylidene fluoride membranes. The membranes were then incubated with appropriate antibodies. Finally, the proteins were visualized using an enhanced chemiluminescence detection kit (Pierce, Rockford, IL, USA).

### 2.6 Cellular proliferation assay

Cell proliferation was assessed using a CCK-8 (Dojindo) assay as described earlier [27]. Briefly, cells were seeded into 96-well plates. CCK-8 assay reagent was added to each well and cultured at 37°C for 2 h. Optical density values of the supernatant from each well were then measured in a microplate reader.

### 2.7 Statistical analysis

Statistical analyses were performed using the SPSS software (IBM, Armonk, NY, USA). The values are presented as the mean ± SD and were analyzed by *t* test. A *P* value less than 0.05 was considered statistically significant.

## 3 Results

### 3.1 SphK2 contributes to the adipocyte-induced EOC cell proliferation

**Figure 1:**
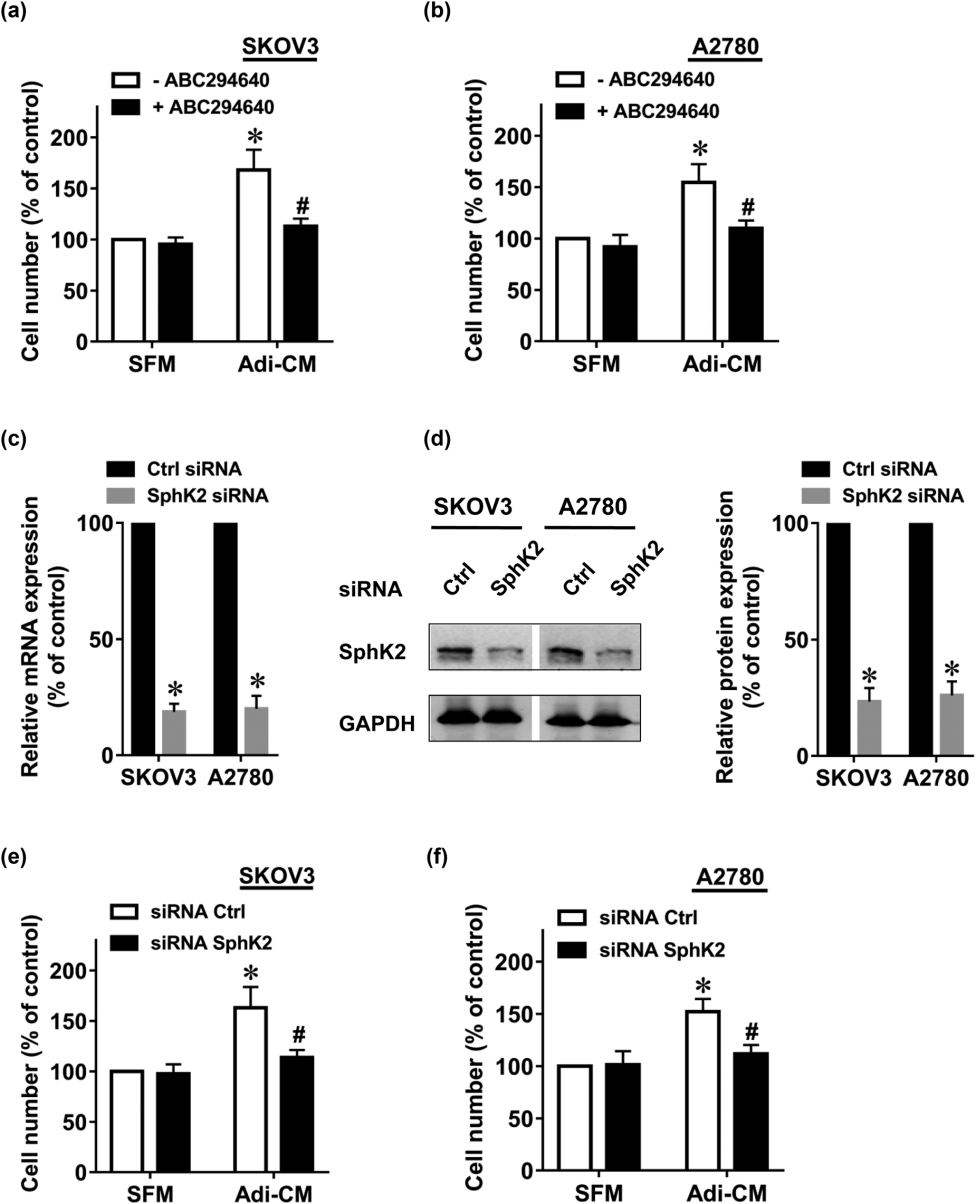
SphK2 inhibition suppresses the adipocyte-induced EOC cell growth. The human EOC cell lines (a) SKOV3 and (b) A2780 were serum-starved overnight and then cultured with SFM or Adi-CM in the presence or absence of ABC294640 (10 μM) for 48 h. Cell proliferation was measured by CCK-8 assay. (c) Twenty-four hours after siRNA transfection, SphK2 mRNA levels were determined by RT-PCR. (d) Forty-eight hours after siRNA transfection, SphK2 protein levels were determined by using Western blot. Densitometric analysis of SphK2 (normalized to GAPDH) is shown on the right. (e) SKOV3 and (f) A2780 cells were transfected with the indicated siRNAs, followed by culture with SFM or Adi-CM for 48 h. Cell proliferation was measured by CCK-8 assays. Molecular weight of SphK2 is 69 kDa, and molecular weight of GAPDH is 36 kDa. Data are mean ± SD. *, *P* < 0.05 vs control; #, *P* < 0.05 vs Adi-CM alone.

To investigate whether the SphK2 pathway participates in the adipocyte-induced EOC proliferation, we used ABC294640 [28], an inhibitor of SphK2. Consistent with earlier reports [3], Adi-CM significantly increased the proliferation rate of EOC cells (Figure 1a and b). Remarkably, ABC294640 significantly inhibited the Adi-CM-induced EOC cell proliferation. As a control, ABC294640 alone did not significantly affect EOC growth (Figure 1a and b). Moreover, SphK2 siRNA significantly inhibited both the mRNA and protein expression levels of SphK2, as shown in Figure 1c and d. SphK2 silencing significantly inhibited the Adi-CM-induced EOC cell proliferation (Figure 1e and f). Collectively, these results suggested that SphK2 contributed to the Adi-CM-induced EOC proliferation.

### 3.2 Adipocytes mediate the activation of SphK2 in EOC cells

**Figure 2:**
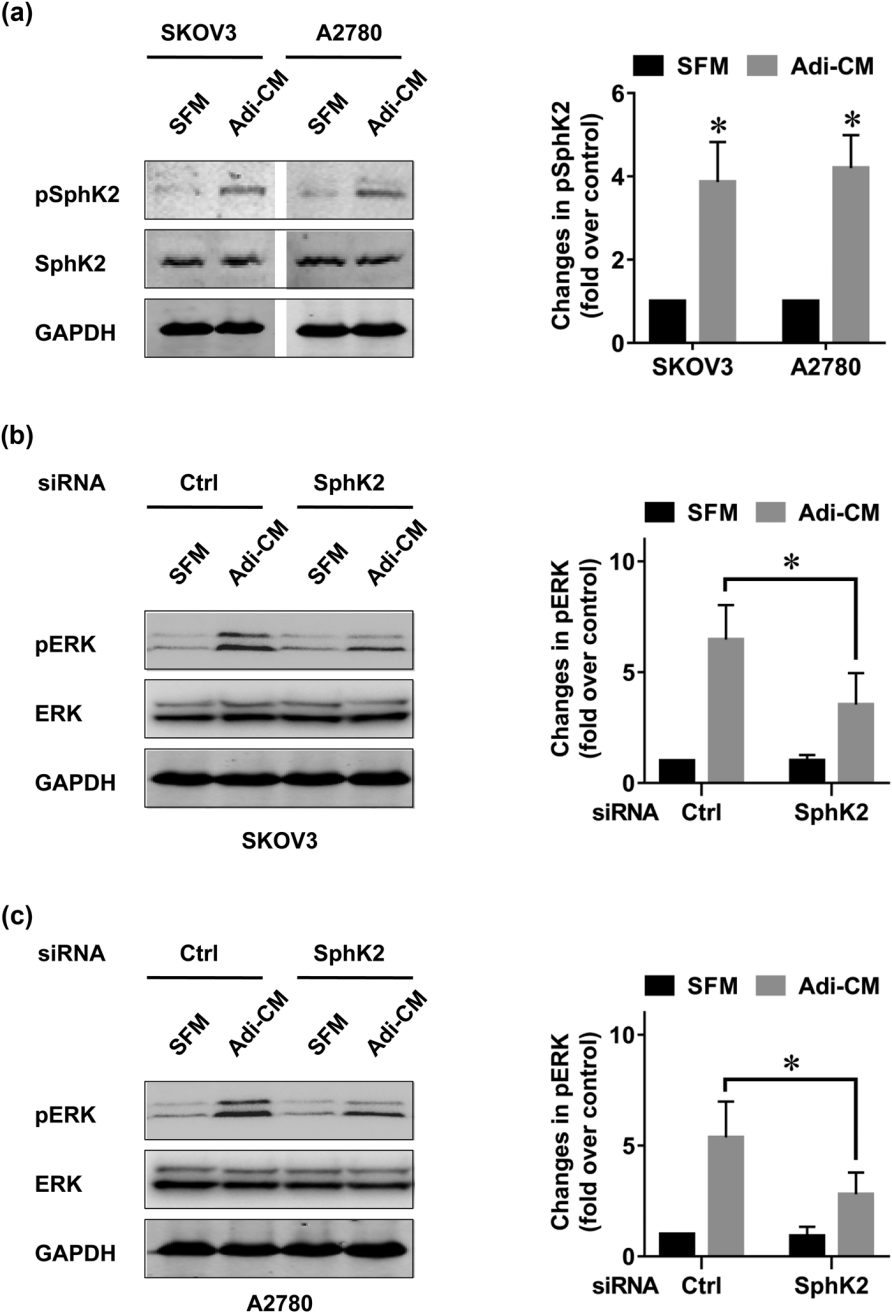
Adipocytes activate the SphK2/ERK pathway in EOC cells. (a) SKOV3 and A2780 cells were cultured with SFM or Adi-CM for 24 h. SphK2 phosphorylation was determined by using Western blot. Densitometric analysis of pSphK2 (normalized to total SphK2) is shown on the right. (b) SKOV3 and (c) A2780 cells were transfected with the indicated siRNAs and cultured with SFM or Adi-CM for 24 h. Total and phosphorylated ERK (pERK) levels were then determined by using Western blot. The right panel shows densitometric analysis of pERK (normalized to total ERK). Molecular weight of pSphK2 is 70 kDa, molecular weight of SphK2 is 69 kDa, molecular weight of GAPDH is 36 kDa, molecular weight of pERK is 42, 44 kDa, and molecular weight of ERK is 42, 44 kDa. Data are the mean ± SD. *, *P* < 0.05.

**Figure 3:**
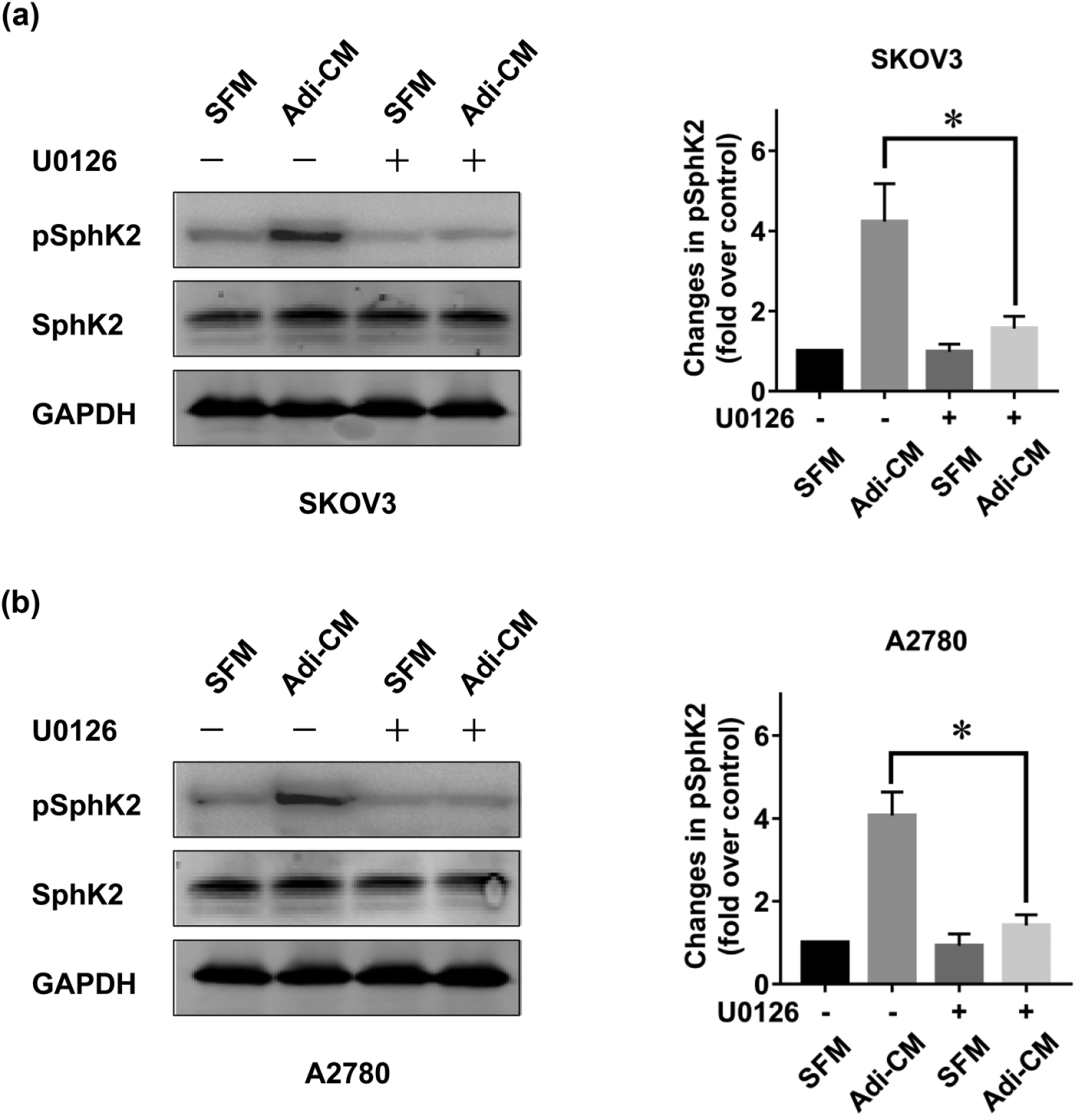
Adipocyte-induced SphK2 activation is ERK dependent. (a) SKOV3 and (b) A2780 cells were serum starved overnight and pretreated with U0126 (5 μM) for 2 h. Cells were then cultured with SFM or Adi-CM for 24 h. Total and phosphorylated SphK2 (pSphK2) levels were then determined by using Western blot. Right panels show the densitometric analysis of pSphK2 (normalized to total SphK2) corresponding to the bands shown in the Western blots. Molecular weight of pSphK2 is 70 kDa, molecular weight of SphK2 is 69 kDa, and molecular weight of GAPDH is 36 kDa. Data are the mean ± SD. *, *P* < 0.05.

As SphK2 is an important enzyme in EOC proliferation, we explored the role of adipocytes in SphK2 activation. It has been reported that SphK2 can be activated by phosphorylation [29]. Therefore, we measured SphK2 phosphorylation in EOC after Adi-CM treatment. The results showed that Adi-CM treatment induced an increase in SphK2 phosphorylation in EOC (Figure 2a). Adi-CM treatment also resulted in increased phosphorylation of ERK (Figure 2b), a key enzyme controlling EOC proliferation [30]. Our earlier study suggested that ERK could be activated through SphK2 [12]. In agreement with this finding, SphK2 blockade significantly inhibited the Adi-CM-induced ERK phosphorylation, which indicated that SphK2 contributed to the Adi-CM-induced ERK activation in EOC. ERK is a key enzyme causing SphK2 activation [29]. Indeed, U0126, an inhibitor of ERK, significantly blocked the adipocyte-induced SphK2 activation (Figure 3a and b).

### 3.3 Adipocyte-induced c-Myc expression in EOC cells occurs partly through the SphK2 pathway

**Figure 4:**
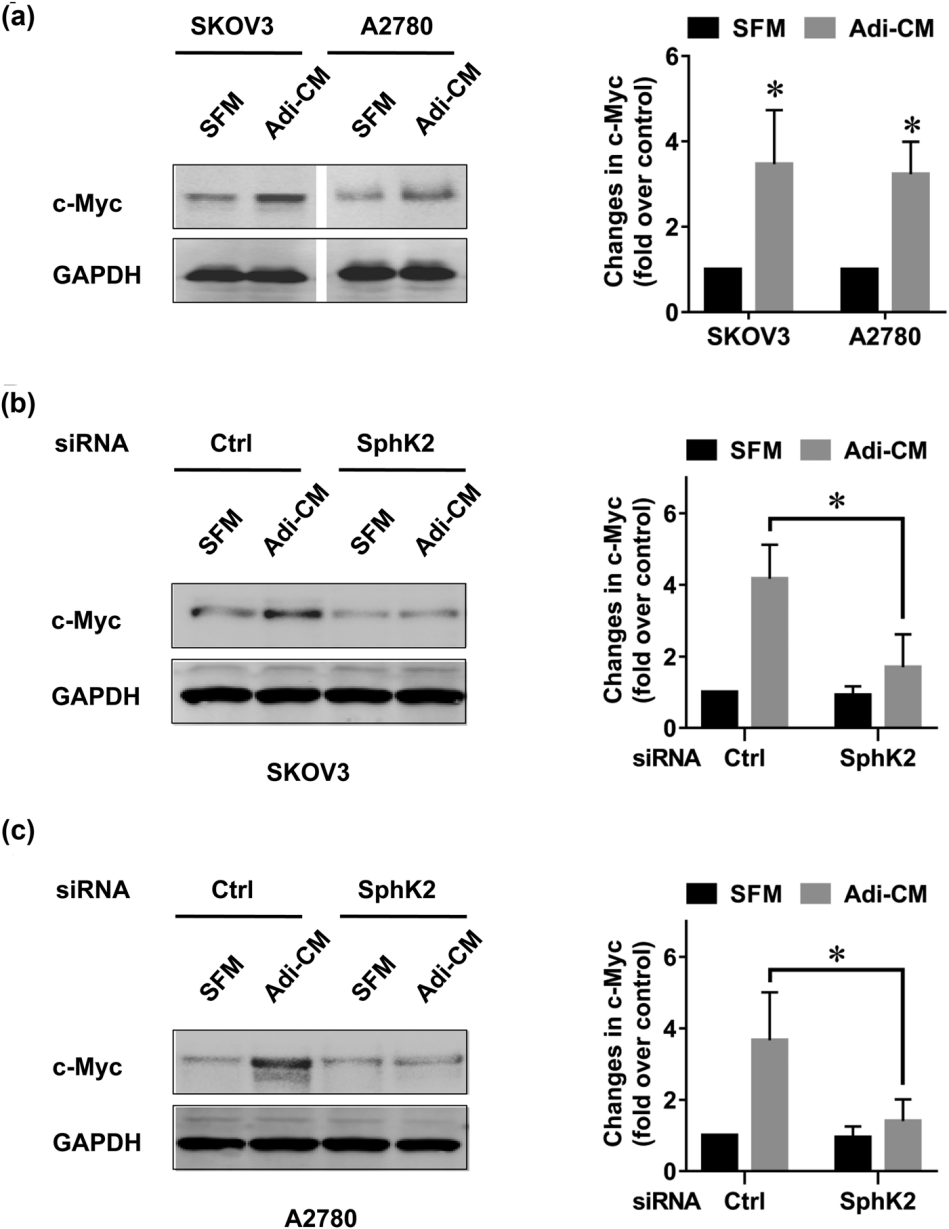
Adipocyte-induced c-Myc expression is partly SphK2 dependent. (a) SKOV3 and A2780 cells were cultured with SFM or Adi-CM for 24 h. c-Myc expression level was determined by using Western blot. Densitometric analysis of c-Myc is shown on the right. (b) SKOV3 and (c) A2780 cells were transfected with the indicated siRNAs and cultured with SFM or Adi-CM for 24 h. c-Myc levels were then determined by using Western blot. Right panel shows the densitometric analysis of c-Myc. Molecular weight of c-Myc is 57 kDa and molecular weight of GAPDH is 36 kDa. Data are the mean ± SD. *, *P* < 0.05.

It is well established that c-Myc is a key mediator of EOC proliferation [31]. Therefore, we detected the expression level of c-Myc and confirmed that Adi-CM treatment significantly increased the c-Myc protein level in EOC cells (Figure 4a). Our earlier studies indicated that c-Myc could be regulated by SphK2 in EOC cells [27]. To determine whether adipocytes induced c-Myc expression in EOC through the SphK2 pathway, we tested the expression level of c-Myc after SphK2 blockade. As expected, SphK2 silencing by siRNA significantly inhibited the Adi-CM-induced c-Myc expression in the two EOC cell lines (Figure 4b and c). Together, these results suggested that adipocytes could activate the c-Myc pathway in EOC cells. The adipocyte-induced c-Myc expression was partly SphK2 dependent.

## 4 Discussion

In this study, we unraveled a previously unrecognized role of SphK2 in mediating the growth-promoting functions of adipocytes in EOC. SphK2, an enzyme that exhibits controversial roles in the regulation of cell growth, is responsible for the adipocyte-induced EOC proliferation. Moreover, SphK2 contributes to the adipocyte-induced ERK and c-Myc pathway activation, both of which are well recognized as key signals that facilitate EOC growth. These results suggested that SphK2 could be a new target for the management of EOC.

Ovarian cancer cells preferentially proliferate in the omentum, an organ primarily composed of adipocytes [3]. Indeed, omental metastases typically represent the largest tumor in the abdominal cavities of women with ovarian cancer. Moreover, adipocytes were reported to promote EOC growth both *in vitro* and *in vivo* [3]. Therefore, understanding the mechanisms involved in the adipocyte-promoted EOC growth is an important research topic. Our results demonstrated that SphK2 was not only activated by adipocytes but also responsible for the adipocyte-induced EOC growth. Adipocyte culture medium was able to stimulate the phosphorylation of SphK2 in EOC cells. Moreover, drug inhibition of SphK2 significantly suppressed the adipocyte-dependent EOC growth. Furthermore, the siRNA-mediated knockdown of SphK2 resulted in a significant suppression of the adipocyte-promoted cell growth, suggesting an important role of SphK2 in the growth-promoting action of adipocytes in EOC cells. This is consistent with our earlier study showing that SphK2 is important for the follicle-stimulating hormone-induced EOC growth [12]. However, studies have indicated the proapoptotic effect of SphK2 on certain cell types [20,32]. Therefore, SphK2 regulates cell growth in a highly cell type-specific fashion. The underlying mechanisms of this cell type-dependent effect of SphK2 remain to be addressed. The SphK/S1P pathway was also reported to play potential roles in the development of drug resistance. For example, targeting SphK2 reversed the acquired resistance to regorafenib in hepatocellular carcinoma [33]. Tamoxifen-resistant breast cancer cells showed increased levels of SphK expression and activity [34]. S1P receptor expression levels were influenced by tamoxifen treatment in breast cancer cells [35]. Our results showed that adipocytes could induce the activation of SphK in EOC cells. Therefore, further studies are warranted to explore the roles of adipocytes in the development of EOC drug resistance. In addition to EOC cells, SphK/S1P signaling could also regulate endothelial cell functions [36,37], such as cell proliferation, migration, and survival. These processes in endothelial cells are essential components of angiogenesis [37]. Therefore, adipocytes may also provide nutrition and oxygen for sustained EOC growth by activating SphK in endothelial cells and inducing angiogenesis.

The ERK pathway is well recognized as a critical signaling pathway that facilitates EOC growth [38,39]. For instance, ERK signaling is constitutively active in EOC cells [30]. Downregulation of the ERK pathway could lead to the complete suppression of EOC proliferation [30]. Moreover, a variety of cytokines and growth factors have been shown to promote EOC growth by activating ERK [40,41]. As important secretory cells, adipocytes release a variety of adipokines, including leptin, IL-8, and IL-6 [42]. Many of these adipokines could promote EOC growth by activating the ERK pathway [43,44]. Indeed, exposure of EOC cells to Adi-CM resulted in increased ERK phosphorylation. We previously found that SphK2 is an important regulator of ERK [12]. Having demonstrated the ability of adipocytes to cause SphK2 activation in EOC, a potential role of SphK2 in the adipocyte-induced ERK activation was suggested. As expected, SphK2 blockade by siRNA significantly inhibited ERK phosphorylation induced by adipocytes. This result indicated that SphK2 plays an important role in mediating the adipocyte-induced ERK activation in EOC. Earlier studies indicated that ERK is a key enzyme that mediates SphK2 phosphorylation [29]. Consistent with this finding, the treatment of EOC cells with U0126, an inhibitor of ERK signaling, significantly blocked the adipocyte-induced ERK phosphorylation. Collectively, these data indicated that ERK resided both upstream and downstream of SphK2, propagating a positive feedback loop.

Another new finding of this study is that the adipocyte-induced c-Myc expression is partly SphK2 dependent. As an important oncogene, c-Myc has been reported to be a crucial mediator of EOC progression. The disease-free survival and OS of ovarian cancer patients were decreased with high c-Myc mRNA levels [45]. Moreover, c-Myc silencing significantly inhibited the growth of EOC cells [31,45]. Given the importance of c-Myc in EOC growth, we examined the effect of adipocytes on the expression of c-Myc. We found that adipocyte CM significantly increased the c-Myc protein expression level in EOC cells, which may participate in the adipocyte-induced EOC growth. Earlier studies have shown that SphK2 regulates c-Myc in a number of cancer cells [46,47]. Our recent studies also found that SphK2 inhibition downregulates c-Myc expression in EOC [27]. Consistent with these findings, SphK2 blockade by siRNA significantly inhibited the adipocyte-induced c-Myc expression. These results suggested that adipocyte-mediated c-Myc expression was partly mediated through SphK2. However, the exact mechanism of the adipocyte-induced c-Myc expression is not yet clear. c-Myc could be targeted by other adipocyte-regulated pathways, such as the SphK1 [16,48], AKT [25,49], and ERK [23,50] pathways. In addition, c-Myc could also be activated by adipocyte-secreted factors, such as estrogen [51,52], interleukin-6 [3,53], and interleukin-8 [3,54]. Therefore, we speculate that adipocytes can also induce c-Myc expression through SphK2-independent pathways and some unknown signaling pathways. Moreover, SphK1 and SphK2 may play similar roles in mediating the adipocyte-induced c-Myc expression in EOC cells. These points need further investigation.

The present study has several limitations. First, the experiment was only performed in EOC cell lines. This would be improved by verification of the key results in mouse ovarian cancer models. Second, the mechanisms by which adipocytes activate the SphK2 pathway in EOC cells need to be further explored. Third, adipocytes may also affect tumor growth by acting on endothelial cells, and their mechanism needs to be studied in the future. Finally, SphK was also reported to play other roles in EOC progression, such as invasion and angiogenesis. Whether the adipocyte-induced SphK activation affects EOC metastasis and vascularization needs further study.
